# Nationwide survey of patients’ and doctors’ perceptions of what is needed in doctor - patient communication in a Southeast Asian context

**DOI:** 10.1186/s12913-020-05803-4

**Published:** 2020-10-14

**Authors:** Trung Quang Tran, A. J. J. A. Scherpbier, Jan van Dalen, Dung Do Van, Elaine Pamela Wright

**Affiliations:** 1 University of Medicine and Pharmacy at Ho Chi Minh City, Ho Chi Minh City, Vietnam; 2 Maastricht University, Maastricht, The Netherlands; 3 Guelph International Health Consulting, Amsterdam, The Netherlands

**Keywords:** Doctor-patient communication, Partnership model, Perception, Culture, Asia, Training, Vietnam

## Abstract

**Background:**

Asian countries are making efforts to apply the partnership model in doctor-patient communication that has been used effectively in Western countries. However, notable differences between Western and Asian cultures, especially the acceptance of a hierarchical order and little attention to individuality in Asian cultures, could mean that the application of the partnership model in Vietnam requires adaptation.

The study aimed to investigate whether communication models used in the Western world are appropriate in Southeast Asia, and to identify key items in doctor-patient communication that should be included in a doctor-patient communication model for training in Vietnam.

**Methods:**

In six provinces, collaborating medical schools collected data from 480 patients using face-to-face surveys with a structured guideline following a consultation session, and from 473 doctors using a cross-sectional survey on how they usually conduct consultation sessions with patients. Data collection tools covered a list of communication skills based on Western models, adapted to fit with local legislation.

Using logistic regression, we examined whether doctor patient communication items and other factors were predictors of patient satisfaction.

**Results:**

Both patients and doctors considered most elements in the list necessary for good doctor-patient communication. Both also felt that while actual communication was generally good, there was also room for improvement. Furthermore, the doctors had higher expectations than did the patients. Four items in the Western model for doctor-patient communication, all promoting the partnership relation between them, appeared to have lower priority for both patients and doctors in Vietnam.

**Conclusion:**

The communication model used in the Western world could be applied in Vietnam with minor adaptations. Increasing patients’ understanding of their partner role needs to be considered. The implications for medical training in universities are to focus first on the key skills perceived as needing to be strengthened by both doctors and patients. In the longer term, all of these items should be included in the training to prepare for the future.

## Background

Doctor-patient communication is one of the most essential dynamics in health care, affecting the course of patient care and patient satisfaction [1–5]. Although technical skills may receive more attention in physician training, communication plays an essential role in practice.

A standard communication structure for a medical interview is described in the Calgary-Cambridge Observation Guide [2], which has been an effective model for communication skills in medical interviews and has strongly influenced the development of medical communication skills training programs [6, 7]. In this “partnership” model, which has been effectively applied in Western countries, the patient is actively involved in the consultation, and doctor and patient are equal [8]. Some Asian countries are trying to shift their doctor-patient communication model toward this partnership model; however, application of a Western model to Asian communities may require it to be adapted to fit local context [9–12].

The Western communication model improves patient care and patient satisfaction. Patient participation in medical encounters depends on a complex interplay of influencing factors, including ethnicity [11, 13–16]. Hofstede, a pioneering researcher on cross-cultural communication and organization, who developed the Cultural Dimensions Theory and Country Comparison Tool [17], reported notable differences between Western and Asian cultures. Using this tool, Vietnam scores highest on the dimension “Power distance” and lowest on the dimension “Individualism,” showing that Vietnamese people accept a hierarchical order in which everybody has a place without further justification, and pay less attention to the individual than to the group. In contrast, in Western countries the “Individualism” score is higher than “Power distance,” meaning that equal respect and individual rights are expected and deserved [18].

In a strongly hierarchical society, the respect for someone who is perceived to be of higher social status strongly influences communication [19]. Doctors are considered to have a higher position than their patients and they recognize that [19, 20]. In addition, patients accept their lower social position; they do not have expectations beyond that [18], and do not think of doctors as their partners [10].

A society with a low score on individualism is a collectivist society where there is a high preference for a strongly defined social framework in which individuals are expected to conform to the ideals of the society [18]. In collectivist societies, self-control is a core value. Vietnamese people tend to be reserved and modest; emotions are typically kept to oneself, whereas expressions of disagreement that may irritate or offend are avoided [20] With such cultural characteristics, applying the partnership communication model in Vietnam may require compromise. For example, a study in Sri Lanka found that fewer than 50% of patients expected doctors to introduce themselves, to thank them, or to discuss available treatment options with them [9].

Although Hofstede’s theories are often seen as the most comprehensive and relevant study of cultural differences [21], there has been criticism of his approach, such as assigning the results from one company to their entire nation’s overall scores, and using nations as units to examine cultural differences [22]. A study replicating Hofstede’s approach with a student population at Stenden University [23] showed that the dimensions “Power Distance” and “Individualism” of Chinese students did differ from Western students, but the differences were smaller than in Hofstede’s report. It was also suggested that work-related cultural values in a specific culture can change over time [24].

In Vietnam, there is not yet a list of required clinical communication skills for medical doctors. The competency standards for general practitioners state: “General practitioners shall have the ability to communicate effectively with patients, their family and the community,” and note two related standards: “Have the ability to create a friendly, cooperative and trustful relationship with patients, their family and the community,” and “Effective communication.” These two standards have nine criteria which reflect the partnership model in doctor-patient communication [25].

There are also Government regulations on communication in medical facilities [26] which guide activities of health workers and include most but not all standard activities in doctor-patient communication. The regulations reflect the partnership model, with the general regulation: “Communication in medical examination and treatment facilities is expressed verbally and by cultural attitudes and behaviors; visiting people are the consumers of services of medical examination and treatment facilities and are treated equally and politely.” Implementing these standards and regulations in training for doctor-patient communication is a challenge in Vietnam, because of the lack of a formal Vietnamese doctor-patient communication model.

Based on these government regulations and the available material on doctor-patient communication (mostly from Western countries), each university had developed its training and assessment material in its specific way. A typical Vietnamese doctor-patient communication model is needed to provide a mandate to introduce the partnership model more systematically into medical education throughout the country.

This study aimed to identify the essential items in communication that doctors should perform during a consultation applying the partnership model; the results will guide the application of the partnership model in training doctor patient communication. We expected that doctors and patients would value most of activities from the Western doctor-patient communication model, but that selected activities related to building a relationship (but not directly related to the patient’s health) may have lower priority for patients and/or doctors.

## Methods

Doctors and patients are both partners in the communication. When investigating which parts of the Western communication model may be applicable in Asia, both the patients’ expectations and the doctors’ willingness and capacity to meet those expectations should be explored. Gender, age, employment, setting, and time available for the consultation may influence patients’ expectations and satisfaction [10, 12, 19, 27–30]. Because they may influence the communication, we recorded these potentially confounding variables, to support interpretation of the results.

### Study design

The data were collected using face-to-face surveys with 480 patients and a cross-sectional survey among 473 doctors in six Vietnamese cities, implemented by six of the eight oldest medical schools in Vietnam. To reduce bias caused by patients’ life experience, we asked patients about the consultation they had just finished, not doctor-patient communication in general. Because we invited doctors to complete the questionnaire when they had time, we could only ask them about doctor-patient communication in general. Therefore, answers from patients and doctors could not be directly compared.

### Study participants and data collection process

The study employed a nationwide stratified sample of participants. Teaching staff in six medical universities in six provinces/cities with universities implemented it: two in the North, two in the Center and two in the South. One in the North and one in the South are in major cities, while the others are in rural provinces. The data were collected from both urban hospitals and district health centers, none linked directly with the universities. Since district health centers have fewer doctors and patients than do city hospitals, we selected one urban hospital and two district health centers in each province/city as research sites, based on their order in the list of health facilities and using random number lists generated in Excel. We also used random number lists to select 40 medical doctors and 40 patients at each hospital and 20 medical doctors and 20 patients at each district health center to participate in this study. The doctors worked in outpatient services where they would be expected to use all the communication activities. The patients had just finished a consultation at an outpatient service. Patients and doctors were compensated for their time, receiving around two euros. The research assistants were six groups of four medical teaching staff who had participated in developing the questionnaires and a guide for the face-to-face survey. They were trained in a 3-h session to ensure consistent use of the guide. Research assistants performed the face-to-face surveys and recorded patients’ responses, but did not probe for further qualitative information, so that information from the different locations could be standardized.

### Materials

Representatives from the six medical universities involved in communication skills training had collaborated during a workshop to reach consensus on a questionnaire, based on the theoretical background of Kurtz et al. and the Calgary-Cambridge Observation Guide [2, 31]. The MAAS Global checklist to assess communication skills [32] was used as a reference, as were regulations on communication from the Ministry of Health in Vietnam [26]. Working towards an appropriate model for teaching, we developed a standard tool that listed required communication skills. Using this tool, we investigated the perceptions and expectations of patients and doctors on the communication between them. Since patients are the customers of the service, in this study we paid attention to patients’ perceptions, and explored factors that could potentially affect patients’ perceptions and expectations, as well as items related to their satisfaction. We aimed to obtain information that would guide trainers in selecting key items to be included in doctor-patient communication training.

The doctor also has a crucial role as a partner in the communication. We compared the expectations of patients and doctors to identify any differences that could be addressed during doctors’ training.

#### Doctors

Doctors were asked to complete a two-part questionnaire: (The Questionnaire for doctor survey developed for this study is provided as Additional File 1.)

- Part 1 included 22 activities that may be used by the doctor in communication with patients in the categories: Introduction, Exploration, Examination, Information, Discussion, and Closure. For each activity, doctors were asked first whether they usually performed it, and second, whether they thought they should perform it and would plan to in future for a better communication model. Each question had Yes and No as answer categories.
- Part 2 related to demographic information. Doctors were also asked how much time they used for an average consultation and how much they would need under ideal conditions.

We used an expert panel to increase content validity, and piloted the questionnaire, asking participants to give examples. The reliability of the doctors’ questionnaire using the test-retest method was 0.76 (Pearson correlation coefficient).

#### Patients

As described, this questionnaire served as a guide for a face-to-face survey with patients exiting a consultation (The Form for patient face-to-face survey developed for this study is provided as Additional File 2); teaching staff of local medical universities conducted the survey.

To eliminate any hesitation to be critical, which might negatively impact their treatments, and to encourage patients to participate, the research assistants emphasized that they were not hospital staff and that patients’ responses would remain anonymous. The study was verbally introduced to participants as a national research to explore the perceptions of doctors and patients on their communication at the present time, and their expectations of better communication in the future. By responding, the participants would help the medical universities improve their training quality. The research did not aim to evaluate either the individual doctor or the individual health facility.

Patients were asked to give verbal consent to participate. If they agreed, information on their gender, age, and employment were recorded, as well as the level of the facility. Then patients were asked about their satisfaction with the consultation in general as well as with their communication with the doctor. For each question, patients were asked to choose one of four choices, ranging from very unsatisfied to very satisfied. Additionally, they were asked their opinion on each item of communication on the list of activities in the questionnaire for doctors.

The data collection instruments were pre-tested in a population near a medical university. The pilot revealed that results were unreliable if patients completed the forms without guidance, because they may misunderstand the wording. Some questions were reformulated to be clearer, and the revised questionnaire was administered by research assistants who recorded patients’ responses after making sure that the question was understood. During pilot testing, we found that patients did not clearly understand two of the doctors’ activities on the list: item 12: “doctor asks patient about their knowledge and attitude concerning their disease before giving information” and item 16: “doctor considers patient’s reaction to information provided.” Therefore, these two activities were excluded from the final question list for patients. The reliability of the face-to-face survey form for patients using the test-retest method was 0.73 (Pearson correlation coefficient).

The questionnaire was developed and administered in Vietnamese. The final version was translated into English and edited by the native English-speaking author in consultation with the Vietnamese author.

#### Data analysis

The data from both sets of questionnaires were first analyzed descriptively using SPSS. The categorical variables were reported as frequencies and percentages and compared by age group, sex, employment, or level of health facility using regression analysis. Normally or near-normally distributed variables were expressed as means with standard deviations and compared by Student’s t-test. Non-normally distributed variables were reported as medians with interquartile ranges and compared using the Kruskal-Wallis test and McNemar’s test for paired nominal data. In this study, a threshold *p*-value of 0.05 indicates significance, and a frequency higher than 50% was considered as majority. Stepwise multivariate logistic regression analysis was used to find the most independent items in the doctor-patient communication affecting patient satisfaction. The inclusion and exclusion threshold of the stepwise method were set at *p*-value of 0.10.

#### Ethical considerations

The patients were recruited by invitation, and most invitations (95.4%) were accepted. The face-to-face survey took place in private spaces in the hospitals and district heath centers. Patients were presented verbally with the aim of the study and were informed that they could refuse any questions or could stop the face-to-face survey at any time if they wished without giving an explanation. All participants gave verbal consent to participation.

More than 98% of doctors invited to complete the survey agreed; forms were returned to the Director of Nursing in the outpatient clinic, who forwarded them to the researchers.

The research protocol was approved by the Scientific Committee of the Ho Chi Minh City University of Medicine and Pharmacy, covering both technical and ethical aspects. The relevant authorities in each health facility also agreed to host the study.

## Results

### Patients’ perceptions of doctors’ communication

Most of the patients reported finding their *consultation* satisfactory (75.2%). While one-fifth of the respondents were very satisfied (20.6%), only 4.2% were not satisfied and none was very unsatisfied. When asked about their general satisfaction with the doctors’ *communication*, nearly all patients were satisfied (75.6%) or very satisfied (19.2%), and no one was very unsatisfied.

The patients were then asked about a list of specific activities that may be performed by the doctor during the consultation. For each activity, patients were asked first whether their doctors had performed that activity during the consultation just completed. Then they were asked about their expectations – whether they would like or expect their doctors to perform those specific activities in future consultations.

The patients’ perceptions are presented in Table 1. The items are listed in the order they appeared on the face-to-face survey form. For each activity, the percentages of patients reporting the doctor “performed during last consultation” and the percentages of patients who “expect doctor perform in future” are presented in the same row.

**Table 1:** Patients’ perceptions on performance and need for communication skills (*N* = 480)

	Activities	Performed during the last consultation %	Expect to be Performed in the future %
1	Doctor greets patient	65.6	73.3
2	Doctor introduces him/herself	10.4	37.4
3	Doctor uses the patient’s name in communication	38.4	46.2
4	Doctor listens attentively while patient talks	93.3	98.5
5	Doctor expresses sympathy with patient	86.0	97.1
6	Doctor expresses a positive and encouraging attitude towards patient’s efforts in taking care of health	30.2	60.6
7	Doctor check whether he/she understood exactly what patient said/would like to say	52.9	83.5
8	Doctor asks if there is anything else that patient would like to share.	44.6	81.6
9	Doctor informs the patient what he/she is going to do	63.3	90.6
10	Doctor explains the needs for examination/ test	57.3	89.8
11	Doctor conducts examination in respectful manner	91.9	98.3
12	Doctor informs patient about results of examination	76.9	98.3
13	Doctor informs patient about diagnosis, hypothesis.	62.9	91.2
14	Doctor informs patient about possible prognosis of the disease	58.5	92.1
15	Doctor discusses with patient about treatment methods with advantages and disadvantages of each method	39.4	81.2
16	Doctor summarizes what he/she and patient agreed	46.0	77.7
17	Doctor asks if the patient has any difficulty to follow the treatment course described by the doctor	31.0	77.3
18	Doctor asks patient to repeat main issues in treatment course	26.7	59.6
19	Doctor asks if patient is satisfied with the consultation	15.6	41.2
20	Doctor thanks the patient	12.1	33.5

Note: For all items, the expectations scored significantly higher than the reported performance

For half of the activities, fewer than 50% of patients reported “doctor performed during last consultation.” For all items, the percentage of patients expecting to see those items in future was significantly higher than the percentage reporting that they had been done during their consultation, suggesting that patients wanted doctors to improve on all items. There were four items that less than 50% of patients expected doctors to perform: Doctor introduces him/herself; Doctor uses the patient’s name in communication; Doctor asks if patient is satisfied with the consultation; Doctor thanks the patient.

#### Doctors’ perceptions on doctor-patient communication

Doctors answered a questionnaire that was very similar to questions given to patients, with the addition of the two points that patients had found difficult in pre-testing. They were asked first whether they performed these activities routinely, then whether they thought they should in future. The doctors’ perceptions are presented in Table 2. The items are listed in the same order as they appeared in the questionnaire. For each activity, the percentage of doctors who reported “routinely perform” in the consultations and “expect to perform in the future” are reported in the same row.

**Table 2:** Doctors’ perception of performance of and need for communication activities

	Activities	Routinely perform		Expect to perform in the future	
		N	(%)	N	(%)
1	Doctor greets patient	469	89.3	468	98.7
2	Doctor introduces him/herself	467	30.4	468	74.4
3	Doctor uses the patient’s name in communication	468	41.0	463	60.0
4	Doctor listens attentively while patient talks	467	98.3	464	99.6^a^
5	Doctor expresses sympathy with patient	469	97.2	468	99.6
6	Doctor expresses a positive and encouraging attitude towards patient’s efforts in taking care of health	470	81.3	468	99.6
7	Doctor check whether he/she understood exactly what patient said/would like to say	467	78.4	466	98.7
8	Doctor checks if there is anything else that patient would like to share.	469	88.9	465	98.9
9	Doctor informs the patient what he/she is going to do	469	91.7	467	97.9
10	Doctor explains the needs for examination/test	469	92.8	466	98.5
11	Doctor conducts examination in respectful manner	467	97.9	467	99.6
12	Doctor asks patient about their knowledge and attitude concerning their disease before giving information ^b^	467	67.2	469	95.5
13	Doctor informs patient about results of examination	467	89.1	467	95.3
14	Doctor informs patient about diagnosis, hypothesis.	469	80.2	467	92.9
15	Doctor informs patient about possible prognosis of the disease	467	81.8	466	92.5
16	Doctor consider patient’s reaction to information provided ^b^	465	79.6	465	97.6
17	Doctor discusses with patient about treatment methods with advantages and disadvantages of each method	467	73.7	463	92.7
18	Doctor summarizes what he/she and patient agreed	469	72.3	466	94.6
19	Doctor asks if the patient has any difficulty to follow the treatment course	466	74.7	465	97.4
20	Doctor asks patient to repeat main issues in treatment course	466	30.9	462	77.3
21	Doctor asks if patient is satisfied with the consultation	467	41.3	464	85.1
22	Doctor thanks the patient	466	45.9	465	86.0

^a^ NO significant difference; all other differences between performed and expect to perform were significantly different

^b^Items not included in the patients’ questionnaire

Table 2 shows that five of the 22 activities were not routinely performed by fewer than half of the doctors. For all but one item (number 4,” Doctor listens attentively while patient talks”), the percentage of doctors expecting to perform them in the future was significantly higher than the percentage of those routinely performing them. That one item was reported as routinely done by nearly all doctors and could hardly be improved. On all other items, doctors apparently thought they should improve their communication.

#### Time for each consultation

Doctors’ communication may be influenced by the time available per consultation. They were asked how much time they had for an average consultation and what would be ideal. While doctors said they would need an average of 17.6 (+/− 9.1) minutes per patient, they estimated that they only had 10.8 (+/− 6.6) minutes.

#### Comparison of patients’ and doctors’ expectations

Patients expected improvement on all 20 items, while doctors expected to improve 21/22. However, patients’ expectations for all the items were lower than doctors were. Table 3 shows the five items for which both patients and doctors had the lowest expectations.

**Table 3:** Five items for which both patients and doctors had low expectations

Activities	Patients expected %	Doctors expected %
Doctor thanks the patient	33.5	86
Doctor introduces him/herself	37.4	74.4
Doctor asks if patient is satisfied with the consultation	41.2	85.1
Doctor uses the patient’s name in communication	46.2	60
Doctor asks patient to repeat main issues in treatment course	59.6	77.3

Although the patients had low expectations on all four items, for one (‘Doctor asks patient to repeat main issues in treatment course’), it was above the 50% cutoff for importance. The other four items that most patients did not expect from doctors were still considered important by doctors but were among the five rated lowest by them.

#### Factors potentially affecting patients’ perceptions

To check for potential confounding factors, additional data were collected from patients about their gender, age, and employment. Responses from men and women were similar. Women reported higher frequencies of “doctor performed” than men, but the difference was significant only for the activity “greets patient”. Significantly higher satisfaction scores were seen among respondents over 55 years of age. For employment, few differences were noted. Farmers more often responded “very satisfied”, and industrial workers were more” unsatisfied” with both the consultation and the doctor’s communication. For” doctor greets patient”, retired persons reported the highest frequency, while students reported the lowest. Housewives said that they felt that the doctor “expresses sympathy with patient” more than did students.

The responses from patients exiting a provincial hospital were significantly higher than from a district facility for “Doctor informs the patient what he/she is going to do”. For” Doctor expresses a positive and encouraging attitude towards patient’s efforts in taking care of health”, the results were reversed.

For patients’ expectations of future consultations, no significant differences were found among different groups related to gender, age, employment, or level of facility.

#### Items related to patients’ satisfaction

Analysis of the association between the patients’ perception about the communication actions and their satisfaction with their consultation can clarify what is important to the patients. Logistic regression analysis showed a significant relationship between patients’ general satisfaction with the consultation in general, and with four of the items that were performed by the doctor (Table 4).

**Table 4:** Four items of doctors’ communication significantly related to patients’ general satisfaction with the consultation

Communication activities performed by doctors strongly related to patients’ general satisfaction with the consultation	Sig.	Odds Ratio
Doctor expresses sympathy with patient	.018^a^	3.837
Doctor informs the patient what he/she is going to do	.046^a^	2.745
Doctor conducts examination in respectful manner	.020^a^	3.692
Doctor asks patient to repeat main issues in treatment course	.020^a^	0.283

^a^significantly related

In Table 4, the odds ratio (OR) for the item “Doctor expresses sympathy with patient” was 3.837, which means that patients who reported that their doctor performed this item were 3.837 times more likely to report being satisfied with their consultation than those reporting that the doctor did not perform it. Similarly, the OR were 2.745 and 3.692 times for the items “Doctor informs the patient what he/she is going to do” and “Doctor conducts examination in respectful manner” respectively. For the item, “Doctor asks patient to repeat main issues in treatment course”, the OR of .283 means that this item was inversely related to overall satisfaction with the consultation.

Regarding the relation between the doctors’ performance and the patients’ satisfaction with the doctors’ communication, the results of logistic regression analysis are presented in Table 5.

**Table 5:** Items performed by doctors and significantly related to patients’ satisfaction with doctors’ *communication*

Items performed by doctors that were significantly related to patients’ satisfaction with doctors’ *communication*	Sig.	Odds Ratio
Doctor introduces him/herself	.043^a^	0.267
Doctor listens attentively while patient talks	.002^a^	5.582
Doctor conducts examination in respectful manner	.029^a^	3.363

^a^significantly related

Table 5 shows that two items, “Doctor listens attentively while patient talks”, and “Doctor conducts examination in respectful manner” were significantly related to the patients’ satisfaction with doctors’ communication, while the item “Doctor introduces him/herself” was inversely related with their satisfaction.

## Discussion

Communication between doctor and patient is a key part of a clinical experience [33]. Discussions arise about potential differences in communication practice and expectations in different cultures [13]. When first asked about their satisfaction with the consultation, nearly all patients were either satisfied or very satisfied with both the overall consultation and the doctor’s communication. Robbins et al. reported that patients are most satisfied with consultations when they talk about specific therapeutic interventions, are examined, and receive health education [34]. In our research, most patients reported being satisfied or very satisfied with both the overall consultation as well as with their doctor’s communication. However, when asked about specific communication activities, fewer than 50% of them reported the doctors having performed half of the activities on the list at most. This result suggests that Vietnamese patients were perhaps easily satisfied, and/or had low expectations for the communication with the doctor. On the other hand, the result may have been influenced by putting those questions at the beginning of the face-to-face survey. If we had asked these questions at the end of the survey, after the patients had had an opportunity to review what the doctor had done and what should be done in the future, their overall satisfaction might have been lower.

The four items that less than half of the patients expected the doctor to perform were among the five items that doctors had lowest expectation of performing in future. These items may reflect the relatively little attention to the individual in Asian culture and the acceptance of hierarchy [18]. The patients may place themselves in a lower position than the doctors and limit their expectations. A similar result was found in Sri Lanka (a South Asian country) where less than 50% of patients expected doctors to introduce themselves and to thank the patients [9]. But there were also differences between the Sri Lankan results and the current study results. In Sri Lanka, patients also expected the doctor to decide the most appropriate treatment modality instead of discussing the available treatment options with them. They wanted precise instructions instead of explanations about the disease and the treatment [9]. This may reflect an even stronger hierarchy in Sri Lanka than in Vietnam. This is in support of the findings of Hofstede who reported that the power distance score was higher in Sri Lanka than in Vietnam [18].

Verlinde et al. [35] noted that social differences between doctors and patients could affect communication. Many still follow traditional social rules in which doctors dominate and patients remain passive [36]. Together with better communications training for doctors, increasing the patient’s understanding of their partner role needs to be considered.

The variable” gender” was found to affect only one item: women reported that their doctors” greet patients” more than did men. Women may pay more attention to the greeting, or doctors may be more attentive to women. A few differences were found in satisfaction level and perception by different age groups, employment and with facility level, but expectations were similar for all groups at both facility levels**.**

Most patients in our quantitative study gave low priority to four communication aspects, perhaps because of cultural differences or perhaps because the study methods did not allow for probing to obtain qualitative information.

In a similar hospital-based study among patients in Yemen, patients rated the doctors’ basic communication skills as good, while higher social skills like involving patients in decision-making were considered weak [30]. Among their patients, those above 45 were grouped as” older” and were also more accepting of the doctors’ communication skills, while the younger people were more demanding, similar to the results in our study. Unlike the patients in our study, they found that gender played no significant role in the rating of doctors’ skills [30].

All doctors said they needed more time for consultations. The high SD in their estimates of needed time may be related to specialty or personal speed; however, it could result from the bias of the estimates, since each doctor may have their own way of estimating the average consultation times. In any case, the average perception of the amount of time needed was significantly higher than the time allocated, indicating a time pressure that may force doctors to choose priorities during the consultation.

When we looked at overall patient satisfaction with the consultation or with the doctor’s communication, and its relation to specific items on the communication list, it was revealed that satisfaction was significantly related to items of attentiveness and respect, but two items were notably inversely related to satisfaction. Those were “Doctors introduces him/herself” and “Doctor asks patient to repeat main issues in treatment course.”

“Doctor who introduced him/her” did not seem to harm the patient in communication but if linked with the fact that all doctors said they needed more time for consultations, the patient may think that such activities could waste the valuable but limited time of the consultation especially in the condition that most of the consultation rooms have the name of the doctor and the nurse.

In contrast, because it is not easy for patients to “repeat major problems during treatment”, patients may feel uncomfortable when their doctor asks them to do this. However, by doing this, doctors know for sure whether the patient understands and can remember what to do. The healthcare system must raise patient awareness about the importance of checking mutual understanding and help them get used to it.

It seems that clinicians (and patients) in Vietnam focus on clinical information and examination. However, good medical practice suggests they should maximize therapeutic effects of communication to achieve results through increasing mutual understanding and trust, associated with increased self-efficacy, adherence, social support and improved health [37]. Both doctors and patients in our study recognized a need to strengthen other aspects of communication. However, doctors and patients also agreed that four items on the Western-inspired communication model had lower priority.

The doctors in our study felt confident in their communication skills, while recognizing that they could and should improve in most of the items. The patients’ expectations were less ambitious than the doctors’. An Indonesian report [14] noted that patients wanted doctors to improve their communication skills; patients’ expectations about doctors’ communication were very similar to those reported for Western societies. A U.S. review found that doctors and patients agreed on what constitutes competent communication with patients, but that it often did not happen as expected [37].

### Implications for communication skill training in medical universities

What do these results mean for skills training in medical universities? Firstly, most activities on the list were considered appropriate for doctor-patient communication in Vietnam and were similarly appreciated by both doctors and patients. Also, both clearly recognized that improvements were needed. Claramita and Majoor [38] noted that although they could not detect differences in the actual practice of communication skills between doctors who had or had not been trained in a skills lab, those who had been trained were more aware of their deficiencies and the need to strengthen them.

Claramita et al. [12] considered the relevance of a Western communications approach in a Southeast Asian setting and found that not all items would be appropriate. They did find evidence of interest among patients in a more partnership-type approach. In our study, most patients gave lower priority to four items aimed at fostering the doctor-patient relation, as did most doctors. The role of the social gradient described by Verlinde et al. [35] could explain the reticence on both sides to engage in a more partnership style communication.

Claramita et al. [11] proposed a guideline for training communication skills in Southeast Asia which has similarities with our findings, such as” doctors should express equality”,” invites two-way conversation”,” active listening, facilitation, responding emotion, empathy”. Furthermore, they emphasized the importance of checking understanding. In our study, item “Doctor asks patient to repeat main issues in treatment course”, was inversely related to overall satisfaction with the consultation, but knowing the importance of this element, we have to develop ways to convince patients to become actively involved, and not to ignore that part of the consultation.

#### Limitations

The study was done in outpatient clinics where most patient-doctor contacts would be the first meeting. However, for each case, we do not know whether all communication items were needed, e.g., not all patients may have needed discussion about treatment methods. Some of the responses to survey items might have differed due to factors that were not included in the study, such as whether the patient knew the doctor from a previous visit, and whether these were specialty consultations or for primary care.

Because the design was a cross-sectional survey based on questionnaires, misunderstandings about terminology could lead to incorrect choice of responses. To reduce that problem, the questionnaire was developed and piloted with experts from eight universities before using it in the survey. To collect data from patients, trained research assistants read the questionnaire to each patient and recorded the results, but the doctors completed the questionnaires on their own, making undetected misunderstandings possible. To explore more deeply the expectations of doctors and patients, further studies should include a range of clinical specialties and qualitative research methods should be added.

Very few patients or doctors declined to participate, but those few might have had more negative experiences. Patients and doctors received payment for their time, which could create a positive bias; the amount however was very small. The study investigated communication in a Southeast Asian culture, which could have increased the positive response because of the higher desire for harmony in these cultures. To reduce such bias, participants were informed that they would remain anonymous and the research assistants emphasized that they were not hospital staff, so the patient did not need to hide any criticism.

Asking at the start of the survey about the patient’s general satisfaction could have made that result more positive. If we had put these questions at the end of the survey, after having an opportunity to review in detail what the doctor did and what should be done in the future, the patient’s overall satisfaction might have been lower.

Finally, it may seem a limitation that the doctors’ responses are based on self-reports, which might not reliably reflect their actual performance. The intention, however, was to identify their perceptions about what a good doctor should do; we did not try to evaluate their actual performance.

## Conclusions

Doctors and patients agreed that most of the activities on Western lists for training medical doctors were appropriate. Both groups had similar perceptions about what should be expected under better conditions and both gave lower priority to four skills that are considered important in a Western-style communication promoting relationship. Furthermore, the doctors had higher expectations than the patients. Training of physicians along with improving patients’ understanding of their partner positions must receive attention. It will be important to convince patients to participate actively in their consultation.

The main implication for medical training in universities is that they should focus first on the key skills perceived as needing strengthening by both doctors and patients. The training can be based on Western models with minor adaptations to the local context. In the longer term, all these items should be included, to prepare for the future. If doctors for any reason must omit items during a consultation, they should choose those with lower priority.

Cultural differences affect the position of doctors and patients in doctor-patient communication but applying the partnership model could be implemented by increasing the doctors’ understanding of cultural differences and by increasing patients’ awareness of their partner role.

## Supplementary information

**Additional file 1.**

**Additional file 2.**

## Data Availability

The datasets used and/or analysed during the current study are available from the corresponding author on reasonable request.
