# Supplementary material for: Nationwide survey of patients’ and doctors’ perceptions of what is needed in doctor - patient communication in a Southeast Asian context
Source: BMC Health Serv Res. 2020 Oct 14;20:946. doi: 10.1186/s12913-020-05803-4 (PMC7558741; doi:10.1186/s12913-020-05803-4)
Supplement: Supplementary file 1 — Additional file 1. [file 12913_2020_5803_MOESM1_ESM.doc]

# QUESTIONNAIRE FOR DOCTOR ON DOCTOR- PATIENT COMMUNICATION

This questionnaire is intended to explore the perceptions of doctors on doctor- patient communication at the present time, as well as the doctor’s expectation on a future communication model.

By answering this questionnaire, you will help the medical universities improve their training quality, especially in communication skills. The research does not aim to evaluate either the individual doctor or the individual health facility.

**Part 1**

In this part, for each action (row) you are requested to answer two questions (in two columns A and B):

A. Comment on **existent consultations**: please let us know whether you routinely perform this action or not by ticking **✓** in the Yes or No column.

B. Your **expectation** **in the future**: please let us know whether you expect to perform this in the future consultations or not, by ticking **✓**in in the Yes or No column.

| N | **Action** | A. Routinely perform | | B. Expect to perform in the future | |
| --- | --- | --- | --- | --- | --- |
|  |  | Yes | No | Yes | No |
| 1 | Greeting patient |  |  |  |  |
| 2 | Introducing yourself |  |  |  |  |
| 3 | Using the patient’s name in communication |  |  |  |  |
| 4 | Listening attentively while patient talks |  |  |  |  |
| 5 | Encouraging patient to talk about all their health problems and their concerns in detail |  |  |  |  |
| 6 | Expressing sympathy with patient |  |  |  |  |
| 7 | Doctor expresses a positive and encouraging attitude towards patient’s efforts in taking care of health |  |  |  |  |
| 8 | Checking if you understand exactly what patient would like to say |  |  |  |  |
| 9 | Inform the patient what you are going to do |  |  |  |  |
| 10 | Explain the need for any prescribed examinations/tests |  |  |  |  |
| 11 | Conduct examination with respect for patient |  |  |  |  |
| 12 | Ask patient about their knowledge, attitude concerning their disease before giving information |  |  |  |  |
| 13 | Inform patient about result of examination (signs) |  |  |  |  |
| 14 | Inform patient about diagnosis, hypothesis |  |  |  |  |
| 15 | Inform patient about possible prognosis of the disease |  |  |  |  |
| 16 | Consider patient’s reaction to provided information |  |  |  |  |
| 17 | Discuss with patient about treatment methods with advantages and disadvantages of each method |  |  |  |  |
| 18 | Make a summary of what you and the patient agreed |  |  |  |  |
| 19 | Ask if patient will meet any difficulty in following the treatment course prescribed by the doctor |  |  |  |  |
| 20 | Ask patient to repeat the main issues in the treatment course |  |  |  |  |
| 21 | Ask if the patient is satisfied with the results of the consultation |  |  |  |  |
| 22 | Thank the patient |  |  |  |  |

**Part 2**

23. How much time do you spend for a consultation on average? . . . . . minutes

24. How much time do you think is relevant for an effective consultation? .. . . . . minutes

Personal information of the doctor:

Age ; Male  /Female ; Year of graduation:

Place of work:. . . . . . . . . . . . . . . . . . . . . . . . . . . . . . . . . . . . . . . . . . . . . . . . . . . . . . . . . . . . . . . .

District health center:  City/Provincial hospital:

Thank you for your valuable information.
