# Supplementary material for: Nationwide survey of patients’ and doctors’ perceptions of what is needed in doctor - patient communication in a Southeast Asian context
Source: BMC Health Serv Res. 2020 Oct 14;20:946. doi: 10.1186/s12913-020-05803-4 (PMC7558741; doi:10.1186/s12913-020-05803-4)
Supplement: Supplementary file 2 — Additional file 2. [file 12913_2020_5803_MOESM2_ESM.doc]

**FORM FOR PATIENT FACE-TO-FACE SURVEY**

# ON DOCTOR- PATIENT COMMUNICATION

Date:

Place: . . . . . . . . . . . . . . . . . . . . . . . . . . . . . . . . . . . . . . . . . . . . . . . . . . . . . . . . . .

District health station ; Province/city hospital

### - **Greeting, introduction:** Greeting patient - introduce yourself (Name, place of work)

### **- Introduce the aim of the survey:**

This is a national research to explore the perceptions of patients on doctor- patient communication at the present time, and patient’s expectations of communication in the future. By responding, the participants would help the medical universities improve their training quality.

The research does not aim to evaluate either the individual doctor or the individual health facility.

**- Asking patient’s consent to participate:**

Re-emphasize that “we are research assistants from the universities”, “we would like to invite you to participate in this research, all of information will remain anonymous and you can freely express your opinion.”

**I. Patient data:**

Male  Female ; Age .... ; Profession. . . . . . . . . . . . . . . . . . . . . . . . .

**II. The (level of) satisfaction on this consultation:**

1. How satisfied are you with this consultation? Could you please specify

- Very satisfied

- Satisfied

- Unsatisfied

- Very unsatisfied

2. Are you satisfied with the doctor's communication during this consultation? Could you please specify:

- Very satisfied

- Satisfied

- Unsatisfied

- Very unsatisfied

**III. Patient’s opinion on doctor patient communication:**

Introduce: I will ask some questions about this consultation, could you please let me know if doctor **DID** or **DID NOT DO** each item, *and in your opinion, if that activity* **SHOULD BE DONE** *or* **SHOULD NOT BE DONE in the future (if conditions were better)**

In each row, the research assistant asks the questions in *italics* and considers the appropriate column to be checked*.*

| N |  | **This consultation** | | Expectation for the future | |
| --- | --- | --- | --- | --- | --- |
|  |  | **Did** | **Did Not**  **do** | **Should be done** | **Should not be done** |
| 1 | Doctor greets patient.  *Did the doctor greet you?*  *In the future, do you expect the doctor to greet you?* |  |  |  |  |
| 2 | Doctor introduces himself  *Did the doctor introduce him/herself?*  *In the future, do you expect the doctor to introduce him/herself?* |  |  |  |  |
| 3 | Doctor uses the patient’s name in communication.  *Did the doctor use your name in communication?*  *In the future, do you expect the doctor to use your name in communication?* |  |  |  |  |
| 4 | Doctor listens attentively while patient talk.  *Did the doctor listen attentively while you talked?*  *In the future, do you expect the doctor to attentively listen while you talk?* |  |  |  |  |
| 5 | Doctor expresses sympathy with patient.  *Did the doctor express sympathy with you?*  *In the future, do you expect the doctor to express sympathy with you?* |  |  |  |  |
| 6 | Doctor expresses a positive and encouraging attitude towards patient’s efforts in taking care of health  *Did the doctor express a positive and encouraging attitude towards your efforts in taking care of your health?*  *In the future, do you expect the doctor to express a positive and encouraging attitude towards your efforts in taking care of your health?* |  |  |  |  |
| 7 | Doctor checks whether he/she understood exactly what the patient said/would like to say  *Did the doctor check if he/she understood exactly what you said/would like to say?*  *In the future, do you expect the doctor to check whether he/she understood exactly what you said/would like to say?* |  |  |  |  |
| 8 | Doctor checks if there is anything else that patient would like to share.  *Did the doctor check if there is anything else that you would like to share?*  *In the future, do you expect the doctor to check if there is anything else that you would like to share?* |  |  |  |  |
| 9 | Doctor informs the patient what he/she was going to do.  *Did the doctor inform you what was he/she was going to do?*  *In the future, do you expect the doctor to inform you what are he/she is going to do?* |  |  |  |  |
| 10 | Doctor explains the need for examinations/tests.  *Did the doctor explain the need for examinations and tests?*  *In the future, do you expect the doctor to explain the need for examinations and tests?* |  |  |  |  |
| 11 | Doctor conducts examination in a respectful manner.  *Did the doctor conduct the examination in a respectful manner ? In the future, do you expect the doctor to conduct the examination in a respectful manner?* |  |  |  |  |
| 12 | Doctor informs patient about the results of the examination.  *Did the doctor inform you about the results of the examination?*  *In the future, do you expect the doctor to inform you about the results of the examination?* |  |  |  |  |
| 13 | Doctor informs patient about diagnosis, hypothesis.  *Did the doctor inform you about the diagnosis, hypothesis?*  *In the future, do you expect the doctor to inform you about the diagnosis, hypothesis?* |  |  |  |  |
| 14 | Doctor informs patient about possible prognosis of the disease.  *Did the doctor inform you about possible prognosis?*  *In the future, do you expect the doctor to inform you about the possible prognosis of the disease?* |  |  |  |  |
| 15 | Doctor discusses with patient about treatment methods with  advantages and disadvantages of each method.  *Did the doctor discuss with you about treatment methods with advantages and disadvantages of each method?*  *In the future, do you expect the doctor to discuss with you about treatment methods with advantages and disadvantages of each method?* |  |  |  |  |
| 16 | Doctor summarizes what he/she and patient agreed.  *Did the doctor* *summarize what you and the doctor agreed?*  *In the future, do you expect the doctor to summarize what you and the doctor agreed?* |  |  |  |  |
| 17 | Doctor asks if the patient has any difficulty in following the treatment course.  *Did the doctor ask if you* *have any difficulty in following the treatment course?*  *In the future, do you expect the doctor to ask if you* *have any difficulty in following the treatment course?* |  |  |  |  |
| 18 | Doctor asks patient to repeat main issues in treatment course.  *Did the doctor ask you to repeat the main issues in the treatment course?*  *In the future, do you expect the doctor to ask you to repeat the main issues in the treatment course?* |  |  |  |  |
| 19 | Doctor asks if patient is satisfied with the consultation.  *Did the doctor ask* *if you are satisfied with the consultation?*  *In the future, do you expect to the doctor ask* *if you are satisfied with the consultation?* |  |  |  |  |
| 20 | Doctor thanks to patient.  *Did the doctor thank you?*  *In the future, do you expect the doctor to thank you?* |  |  |  |  |

**Researcher thanks the patient.**
